# Reproductive adaptation in alate adult morphs of the English grain aphid *Sitobion avenae* under starvation stress

**DOI:** 10.1038/s41598-019-38589-5

**Published:** 2019-02-14

**Authors:** Xiangli Xu, Nannan Lv, Qi Shi, Xiangshun Hu, Junxiang Wu

**Affiliations:** Key Laboratory of Northwest Loess Plateau Crop Pest Management of Ministry of Agriculture, College of Plant Protection, Northwest A&F University, No. 3 Taicheng Road, Yangling, Shaanxi 712100 China

## Abstract

Adapting their reproductive physiology is a tactic that insects use in responding to conditions of food unavailability. The present study examined the potential effects of starvation periods on the ovarian development and reproduction of alate adult morphs of *Sitobion avenae* (Fabricius). Morphs both continuously fed and starved aphids contained two telotrophic ovaries, each comprising five ovarioles. As time increase after emergence, the number of offspring produced by the fed aphids increased gradually, whereas the number of embryos in their ovaries decreased gradually. Both the number of mature embryos and the volume of embryos rapidly increased at 24 h after emergence, and then remained at an approximately constant level between 24 and 144 h. Compared to the fed aphids, starved aphids only produced a small number of nymphs, and there was no significant change in the total number of embryos between 24 and 144 h, whereas both the number of mature embryos and volume of embryos increased significantly. Irrespective of starvation period, highly significant relationships between life span and fecundity were found. Adult aphids starved for longer periods presented lower longevity and fecundity, but dead females contained more mature embryos than those starved for shorter periods. These results suggested that, under starvation stress, *S. avenae* tends to invest in the development of larger embryos at the expense of reducing lifespan and future fecundity. This adaptive reproductive strategy under starvation stress could be one of the factors contributing to the successful establishment of new colonies of alate migratory aphids.

## Introduction

Insects develop their ovaries and produce offspring in suitable habitats. However, under adverse conditions such as lack of food, males, social pressure, and oviposition sites, oocytes might be resorbed, rather than developing into eggs^1^. Oocyte resorption is a specific reproductive adaptation widespread in Orthoptera, Lepidoptera, Diptera, Hemiptera, Hymenoptera, Blattaria, and Coleoptera^2–8^. Among ecological, behavioral, and physiological factors, resource stress is a key factor inducing oocyte resorption^1^. Starvation is defined as a failure condition to consume food following an extrinsic restrictions^9^. It rapidly led to oocyte resorption and, when food was provided, oocyte resorption stopped and the ovaries resumed development in *Coccinella septempunctata* (Coleoptera: Coccinellidae), *C. transversoguttata richardsoni* (Coleoptera: Coccinellidae), and *Epilachna niponica* (Coleoptera: Coccinellidae)^8,10^. This reversible dynamic of oocyte resorption and ovary development might be a reproductive adaptation in female adults, enabling them to manage heterogeneous resource conditions^11^. *Pteromalus puparum* (Hymenoptera: Pteromalidae) was observed to release vitellin from the oocytes into the hemolymph after oocyte resorption was induced by starvation^12^. Resorbed oocytes might supply nutrition and energy in order to prolong the survival of starved females, as they can potentially enhance female’ reproduction success in unfavorable environments^3,10,12–14^.

Most insects resorb large oocytes, or oocytes at specific developmental stages in the ovarioles during vitellogenesis, a process in which oocyte resorption is induced^15^. *Megoura viciae* (Hemiptera: Aphididae) and *Acyrtosiphon pisum* (Hemiptera: Aphididae) are viviparous species. Starvation led to resorption of the smallest embryos, but the largest embryos developed continuously in *M. viciae*^16,17^. *Acyrthosiphon pisum* parasitized by *Aphidius smithi* (Hymenoptera: Aphidiidae) also resorbed small embryos^18^. These data indicate that viviparous aphids may adopt an alternative tactic when they are confronted to adverse conditions.

Egg production in female insects is related to the nutrients available. Under starvation stress, resorption of egg chambers at stages 8 and 9 in female flies caused a reduction in the number of their eggs^19^. Starved *Sitobion avenae* (Hemiptera: Aphididae) adults exhibited a significantly lower fecundity than fed adults^20^. Further studies are required in order to confirm this reduction in fecundity is due to resorption of small embryos in aphids.

*Sitobion avenae* is a major pest infecting cereals worldwide; it feeds on phloem sap and transmits viruses^21^. This species prefers temperate climates and cannot overwinter in the north of China. Thus, the establishment of new colonies in this area mainly depends on the migration of alate aphids from south to north; this is supported by suction trap monitoring results^22^. During long-distance dispersal, alates most likely undergo starvation because they lack a suitable host. Often teneral alate adults are starved for different days and fed subsequently, as a result of settling down in their new host plants in the field. Gonadal status is a useful indicator in understanding the reproductive strategy of aphids responding to adverse conditions^23^. To our knowledge, ovarian development has not been examined in alate aphids under starvation stress; nor has its ecological significance in terms of reproductive success. The current study (i) examined ovarian development in the alate aphid *S. avenae* in relation to the presence or absence of food, and (ii) estimated the effect of different starvation periods and subsequent feeding on life span and total fecundity. Based on these results, the reproductive adaptive strategies possibly adopted by migratory aphids during delayed location of the host plants are discussed. The results obtained here will contribute to an understanding of the ecological significance of the successful establishment and continuous reproduction of migratory aphids in a new habitat, particularly regarding their high pest status.

## Results

### Effects of starvation on ovarian morphology

**Figure 1:**
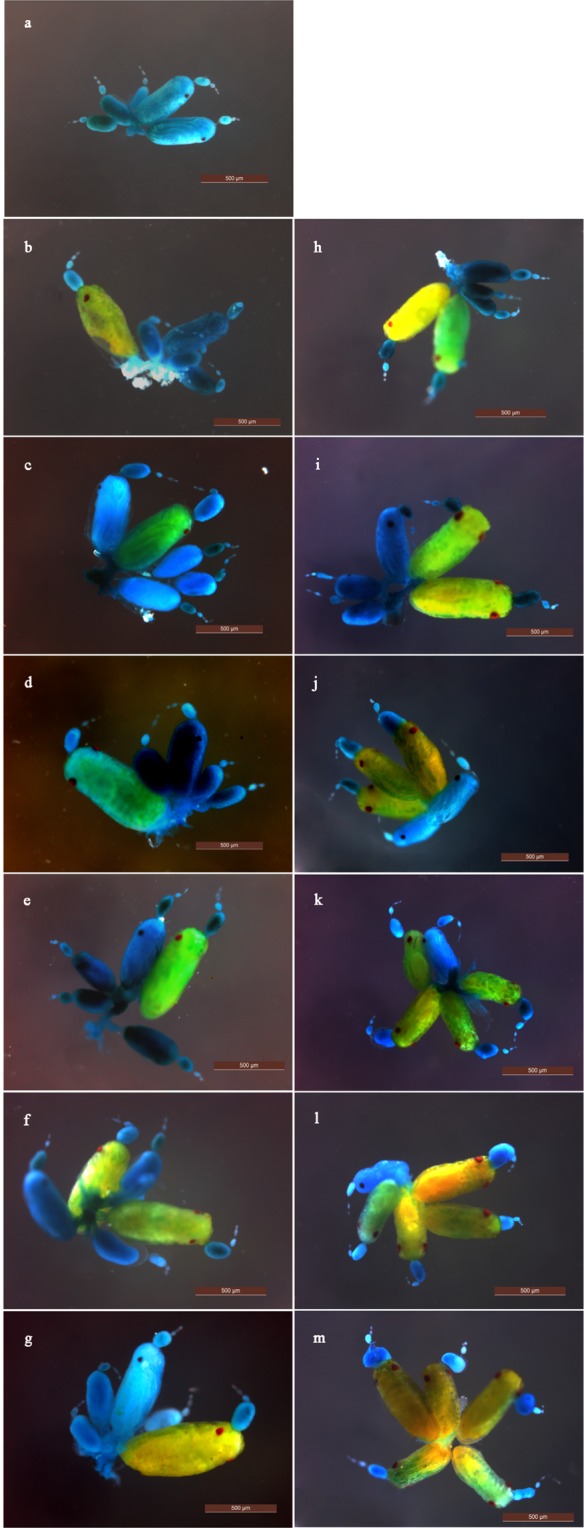
Effects of starvation on the ovarian development of alate *Sitobion avenae* adults under different starvation and feeding treatments. (**a–g**) display ovarian development after 0, 24, 48, 72, 96, 120, and 144 h of feeding, respectively. (**h–m**) display ovarian development after 24, 48, 72, 96, 120, and 144 h of starvation, respectively.

Ovarian morphology in the adult stage subjected to the starvation and feeding treatments is shown in Fig. 1. A few embryos with pigmented eyes were observed in the basal end of ovarioles in newly molted aphids (Fig. 1a). Fed aphids within 144 h of emergence contained two or three embryos with red eyes in the ovary (Fig. 1b–g). Compared to fed aphids, basal embryos within each ovariole of starved aphids continued to develop gradually with increasing starvation time, meaning that there was an increase in the volume of basal embryos and the proportion of embryos with pigmented eyes (Fig. 1h–m). After starvation for 72 h, embryos at the base of each ovariole had almost developed red eyes (Fig. 1j–m). In live adult aphids starved for 144 h, the largest embryos at the base of each ovariole presented with red eyes and appendages, and a few penultimate embryos within the ovarioles occasionally presented red eyespots (Fig. 1m). These results indicated that, as the starvation level increased, larger embryos continued to develop and smaller embryos were still presented.

### Effects of starvation on ovarian development

**Figure 2:**
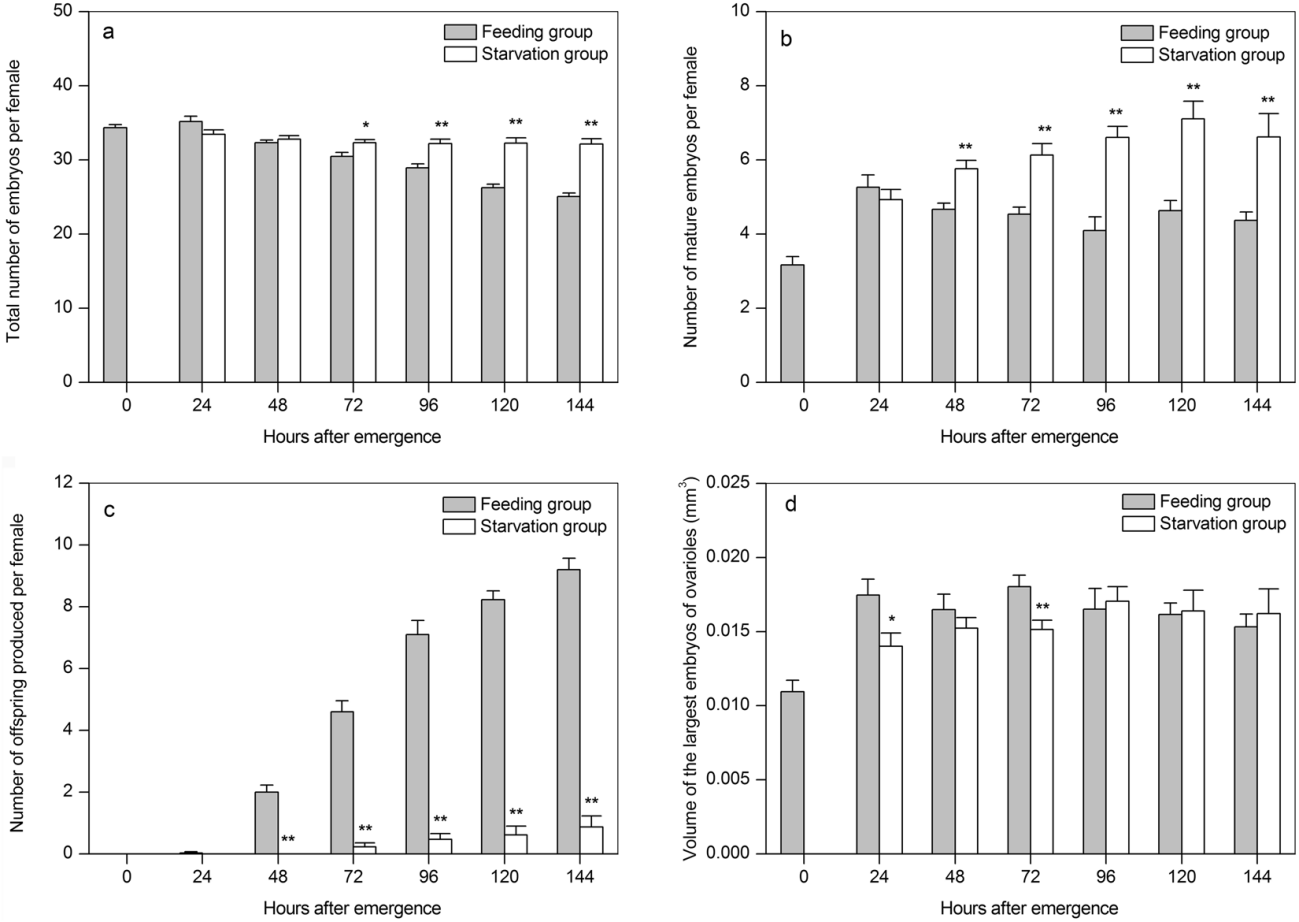
Effects of starvation on the mean (+SE) number of total (**a**), mature (**b**) embryos, and offspring (**c**) produced, and on the mean (+SE) volume of the largest embryos in each ovariole (**d**) of alate *Sitobion avenae* adults under different starvation and feeding treatments. Bars with an asterisk show significant differences between fed and starved aphids at the same stage after emergence (Mann-Whitney U test: *P < 0.05, **P < 0.01, means without an asterisk are not significant at *P* > 0.05).

All aphids dissected contained two telotrophic ovaries, each consisting of five ovarioles, as expected from ovarian developmental symmetry. With increasing time after emergence, there was a gradual increase in the number of offspring produced by the fed aphids (Kruskal–Wallis: H = 151.01, *P* < 0.001, Fig. 2c), whereas the number of embryos retained in their ovaries gradually decreased (Kruskal–Wallis: H = 34.62, *P* < 0.001, Fig. 2a). At 24 h after emergence, both the number of mature embryos and the average volume of the largest embryo in each ovariole rapidly increased. This contrasted with the results for the periods between 24 and 144 h, when their ovaries contained between four and five mature embryos and the largest embryos ranged in volume from 0.015328 ± 0.000870 to 0.018025 ± 0.000793 mm^3^ (ANOVA: *F*_5,174_ = 2.103, *P* = 0.065, Fig. 2b and *F*_5,174_ = 0.888, *P* = 0.490, Fig. 2d, respectively), values which remained approximately constant. During starvation, aphids produced a small number of nymphs, and the total number of embryos showed no significant change with increasing starvation time (ANOVA: *F*_5,137_ = 0.781, *P* = 0.565, Fig. 2a). However, both the number of mature embryos and the average volume of the largest embryo in each ovariole increased significantly (Kruskal–Wallis: H = 69.77, *P* < 0.001, Fig. 2b; ANOVA: *F*_5,137_ = 2.446, *P* = 0.037, Fig. 2d, respectively). Between 48 and 144 h after emergence, the total number of embryos and the number of mature embryos were significantly higher, but number of offspring produced was significantly lower in starved than in fed individuals at the same stage, respectively. Within 72 h of emergence, the largest embryos in the starved aphids presented a greater volume in ovarioles than in the fed aphids at the same stage after emergence. These results further indicated that there was continuous development of the largest embryos with increasing starvation time.

### Relationships between adult mass, life span, and total fecundity

**Figure 3:**
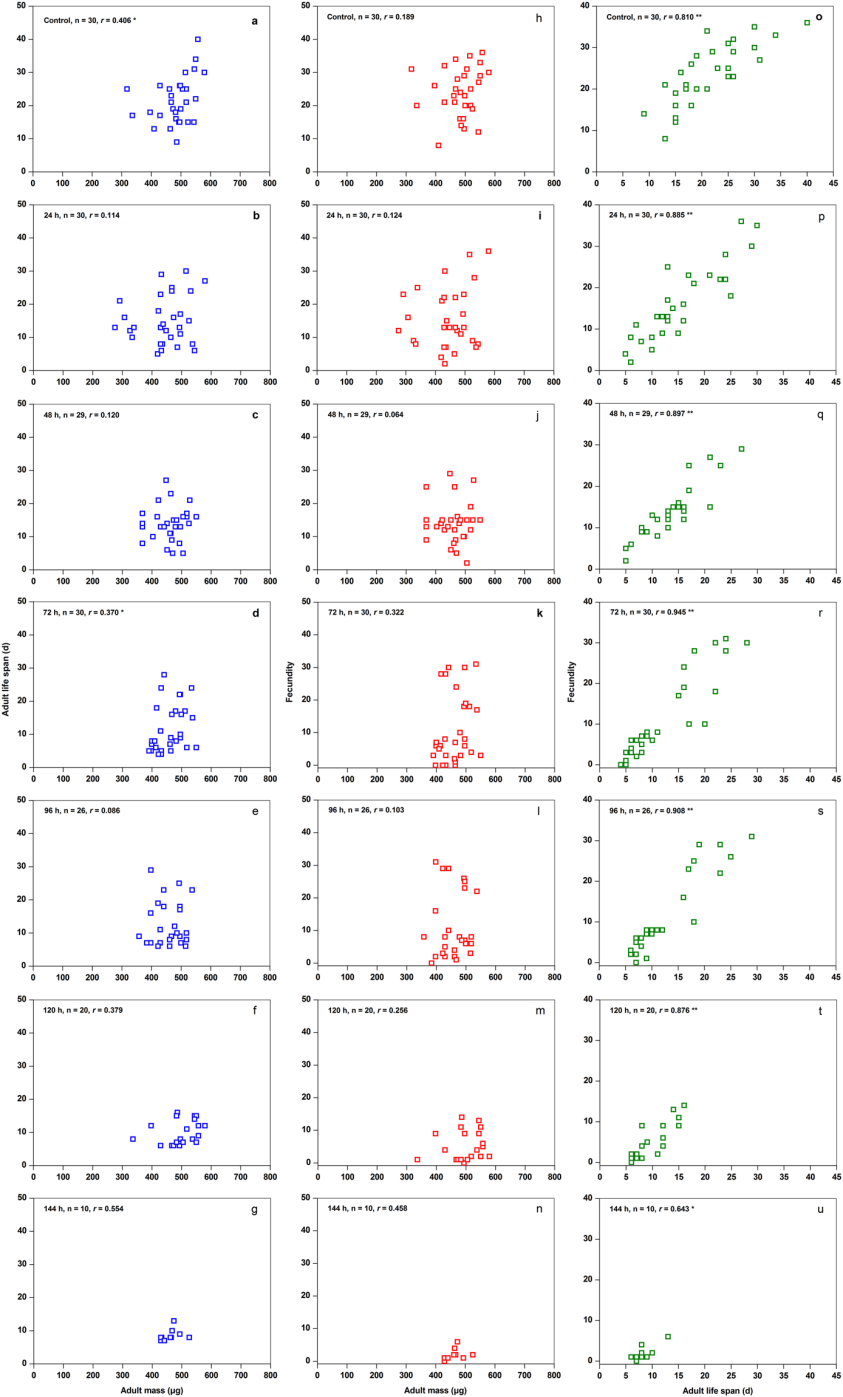
Relationships between adult mass and life span (**a–g**), between adult mass and fecundity (**h–n**), and between life span and fecundity (**o–u**) in alate adults of *Sitobion avenae* under different starvation treatments. Spearman’s correlation coefficients were calculated for the relationships between parameters; ‘*’ correlation significant at *P* < 0.05, ‘**’ correlation significant at *P* < 0.01. Correlations without an asterisk are not significant at *P* > 0.05.

Adult mass was directly related to life span of alate *S. avenae* adults continuously fed and starved for 72 h (Fig. 3a,d), but not directly related to life span of alate *S. avenae* adults starved for 24, 48, 96, 120, and 144 h (Fig. 3b,c,e–g). Adult mass was not directly related to fecundity (Fig. 3h–n) of alate *S. avenae* adults either continuously fed, or starved for 24, 48, 72, 96, 120, and 144 h. However, relationships between life span and fecundity under the different treatments were determined to be highly significant (Fig. 3o–u).

**Figure 4:**
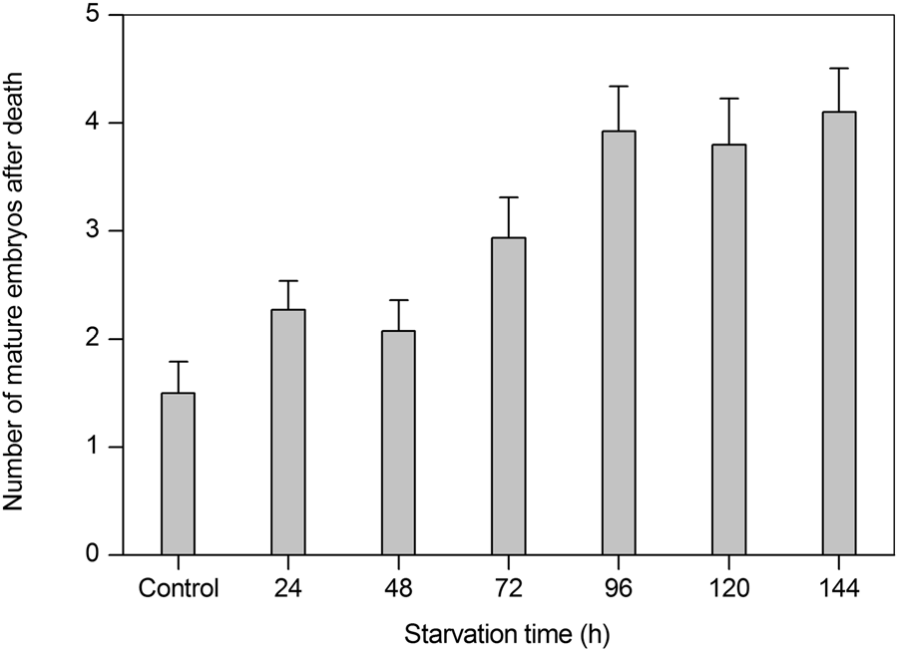
Effects of starvation on the mean (+SE) number of mature embryos in the ovaries of dead alate *Sitobion avenae* females under different starvation treatments.

As starvation time increased, adult life span decreased more rapidly, and dead females contained more mature embryos within their ovaries (Kruskal–Wallis test: H = 33.83, *P* < 0.001, Fig. 4). This result highlighted a marked tendency for females mortality to occur before the release of mature embryos.

## Discussion

Starvation is a common challenge and is an important factor in inducing oosorption^1,24^. Many insect species have been reported to resorb large or specific stages of oocytes in their ovarioles during vitellogenesis under conditions of starvation stress; this leads to a reduction in the number of oocytes and egg load in the ovaries^3,4,6–8,12,13,25–30^. In the present study, the number of mature embryos and the average volume of the largest embryo within each ovariole increased significantly with increasing starvation time (Figs 1 and 2). Thus, larger embryos within ovarioles seem to develop continuously under starvation, in agreement with findings observed in *M. viciae*^16,17^. These data indicate that the resorption of large oocytes is not a generalized reaction in response to starvation stress in insects.

Under starvation stress, the smallest embryos were resorbed in *M. viciae*^16^. This phenomenon was also observed in parasitized *A. pisum* under conditions of nutrient stress^18^. However, the present work revealed that there was no significant decrease in the total number of embryos with increasing starvation time in *S. avenae* (Fig. 2a), indicating that the smallest embryos within ovarioles might not be resorbed. The life span of *S. avenae* decreased with increasing starvation time, but more mature embryos were found in female bodies after death (Fig. 4). Thus, aphids might not benefit from the nutrients provided by the resorption of small embryos intended to ensure female survival. Starvation tolerance and embryo development mainly depend on the lipid content of the fat body^31^. The buffer ability of resorption and the reallocation of the smallest embryos are both extremely limited compared to that in the fat body^32^.

It has long been suggested that apoptosis limits the reproductive potential of females in many animal groups^33^. In the present study, potential fecundity showed no significant reduction tendency based on the total number of embryos found in the gonads under starvation conditions (Fig. 2a). Thus, why did starvation stress lead to a significantly decreased fecundity? Indeed, under starvation stress, the larger embryos within each ovariole of *S. avenae* continued to develop (Fig. 2c). Reproductive investment increases to some extent at the expense of reduced lifespan^34^, which in turn leads to a reduction in reproductive potential. This hypothesis is compatible with the observation of the tendency for mortality to occur before release of the largest mature embryos in alate *S. avenae* adults experiencing starvation stress. The results obtained here indicate that the reduction in *S. avenae* fecundity was strongly correlated with reduced adult life span under starvation stress (Fig. 3o–u).

Ovarian response to starvation, which is vital for reproductive success, is highly variable among insect species under selection pressure^11^. Insects resorb large oocytes in order to store energy and reduce reproductive costs, therefore increasing their survival potential^15^. When resources improve, they resume the reproductive rate in order to enhance their future reproductive efforts^8,35^. Osawa (2005)^29^ report that, under conditions of food availability, *Harmonia axyridis* (Coleoptera: Coccinellidae) presented both ovary development and resorption of oocytes. Moreover, the oosorption rate under starvation conditions was significantly higher than when food was available. This intermediate strategy might be advantageous when there are high fluctuations in food availability. The present study revealed that, under starvation stress, aphids might employ a further strategy, in which large embryos develop continuously in ovarioles, as shown for *S. avenae* (Fig. 2d).

Alate aphids, as host-selective morphs, exhibit a higher resistant to starvation and have a relatively greater number of olfactory sense organs than apterous individuals^20,36^. This phenomenon is beneficial to aphids in finding suitable host plants during dispersal. Reproductive physiology is a means by which insects can respond to resource-poor conditions^14^. *Sitobion avenae* is small and has a short life span. It shows a marked tendency to invest in offspring but not in survival. Under starvation stress, alate aphids display a high mortality rate whereas large embryos in each ovariole show continuous development until reaching a mature stage. However, they are rarely produced offspring. Live aphids survived a starvation treatment can deliver most offspring within a short time after adult aphids are re-fed. Total fecundity and longevity have also been shown to decrease significantly under starvation stress in alate *S. avenae* adults^20^, indicating that aphids safeguard the development of their current embryos at the expense of reducing longevity and future fecundity. Meanwhile, the offsprings from the starved mothers within 24 h of resumption of feeding can successfully develop and reproduce under normal food conditions^37^. Aphids, as a group, can colonize successfully at a new habitat during their migration, which is associated with physiological response to starvation stress. At this time, energy distribution is especially important for its survival and reproduction. Considerable amounts of lipids are depleted in *S. avenae*^38^, *Metopolophium dirhodum* (Hemiptera: Aphididae)^39^, and *M. viciae*^17^ during starvation, indicating that aphids may reallocate energy based on their needs. A part of their resources could be used for flights to find a suitable host plants and another part of resources could be reallocated to larger embryos to ensure a potential offspring. These results suggest that alate *S. avenae* adults are able to show flexibility by taking account of food heterogeneity, and adjusting their commitment to spread their offspring in time and space accordingly. This adaptive strategy is important in their successful reproduction in new habitats during dispersal. Our findings have implications for pest management of *S. avenae*, and provide useful information for future research on insect reproductive adaptation.

## Methods

### Insect rearing

One nymph of *S. avenae* was originally collected from the wheat fields at Yangling, Shaanxi, China (34°18′N, 108°5′E) in 2011 and successively maintained in the laboratory using the water-cultured wheat seedling method^40^. Aphids and wheat seedlings were cultured in a growth chamber at 21 ± 1 °C, 65 ± 10% relative humidity (RH), and a light:dark photoperiod of 16:8 h.

### Experimental setup

Alate aphids molted within the last 6 h were individually weighed using a microbalance (ME36S; Mettler Toledo, Schwerzenbach, Switzerland, 0.001 mg) and adult mass was measured as fresh mass. Subsequently, they were individually placed in Petri dishes containing only with water-saturated absorbent paper and starved for 24, 48, 72, 96, 120, or 144 h (30 individuals per treatment per experiment). Aphids at the same stage after emergence were fed on wheat seedlings as a control. Live aphids survived each treatment were used in the experiments.

### Effects of starvation on ovarian morphology

In order to relate ovarian morphological changes to starvation levels, ovaries were dissected from live aphids that had not produced offspring, and put in a Ringer’s solution on a concave slide, which was then examined under a binocular microscope (SMZ-140-FB; Motic, Shenzhen, China). Aphid starvation time points were for 24, 48, 72, 96, 120, or 144 h. We dissected fed aphids in order to collect their ovaries at the same stage after emergence. Ovaries were stained with 1% bromocresol green buffer for 20 sec, washed three times, each for 1 min in deionized water, and were then transferred to a slide containing one drop of deionized water. The morphology of the ovaries was evaluated according to the different starvation and feeding periods, and photographed using a binocular stereomicroscope (MZS0870; Leica Microsystems, Wetzlar, Germany).

### Effects of starvation on ovarian development

Ovaries were removed from live aphids at the end of each treatment (number of treatments: 30 in 0, 24, 48, 72, 96, 120, or 144 h of feeding, respectively; and 30, 30, 29, 28, 18, and 8, in 24, 48, 72, 96, 120, or 144 h of starvation, respectively). A single drop of 1% bromocresol green was used to facilitate visualization. The number of ovarioles, total and mature embryos, and the average volume of the largest embryos in ovarioles were recorded for each dissected aphid, as these were considered to be the parameters that best describe the status of ovarian development. Embryos were considered as mature if the eyes of the embryo were pigmented^39^. Length (l) and breadth (b) of the largest embryo in each ovariole were measured under a binocular microscope equiped with an ocular micrometer. The accuracy for the length (l) and breadth (b) measures was 0.01 mm. The volume of the largest embryo in each ovariole was calculated using (1 × b^2^ mm^3^) according to Newton and Dixon (1990)^41^.

### Effects of starvation on life span and fecundity

Live aphids at the end of each treatment (number of treatments: 30, 30, 29, 30, 26, 20, and 10, in control (continuously fed), and 24, 48, 72, 96, 120, and 144 h (starvation), respectively) were individually transferred to wheat seedlings, and their daily longevity and reproduction were monitored until their death. Dead females were dissected and the number of mature embryos contained in their bodies was determined under a microscope.

### Statistical analysis

The number and volume of embryos and offspring in the starvation and feeding groups were compared using a one-way analysis of variance (ANOVA). If the data failed to meet ANOVA assumptions by Kolmogorov–Smirnov test, the Kruskal–Wallis method was used to test the differences between the means. Mann-Whitney U tests were used to analyze differences between means of the results from the two treatments at the same stage after emergence. Correlations between adult mass and fecundity, adult mass and life span, and life span and fecundity were calculated using Spearman’s correlations. All data were statistically analyzed using SPSS 13.0 software^42^, considering *P* < 0.05 as the significance threshold.
